# *In vitro* Antioxidant of a Water-Soluble Polysaccharide from *Dendrobium fimhriatum Hook.var.oculatum Hook*

**DOI:** 10.3390/ijms12064068

**Published:** 2011-06-17

**Authors:** Aoxue Luo, Yijun Fan

**Affiliations:** Department of Landscape Plants, Sichuan Agriculture University, Chengdu 611130, China; E-Mail: aoxueluo@hotmail.com

**Keywords:** *Dendrobium fimhriatum Hook.var.oculatum Hook*, polysaccharide, antioxidant activity, *in vitro*

## Abstract

A water-soluble crude polysaccharide (DFHP) obtained from the aqueous extracts of the stem of *Dendrobium fimhriatum Hook.var.oculatum Hook* through hot water extraction followed by ethanol precipitation*,* was found to have an average molecular weight (Mw) of about 209.3 kDa. Monosaccharide analysis revealed that DFHP was composed of mannose, glucose and galactose in a content ratio of 37.52%; 43.16%; 19.32%. The investigation of antioxidant activity *in vitro* showed that DFHP is a potential antioxidant.

## 1. Introduction

*Dendrobium fimhriatum Hook.var.oculatum Hook* (Chinese name “Ma Bian Shi Hu”) is one of the most famous Dendrobium plants in traditional Chinese medicine, it belongs to Orchidaceae, is a precious herbal plant as a therapeutic for nourishing the stomach, promote secretion of saliva, and reduce fever, and it is one of the five species which were specified in the Chinese Phamacopeia (2005). As for Dendrobium species phytochemicals, much research has been carried out on the low molecular compounds, such as bibenzyl [1], phenanthrene [2], and alkaloids [3]. A previous study has proven that the high-molecular-weight compounds such as polysaccharides were major active constituents in *Dendrobium* species [4]. Moreover, most polysaccharides derived from *Dendrobium* species are relatively nontoxic and do not cause significant side effects, which is a major problem associated with immunomodulatory and antioxidant polysaccharides [5], such as *Dendrobium huoshanense* [6], *Dendrobium nobile* Lindl [7], Dendrobium denneanum [8] and *Dendrobium chrysotoxum* Lindl [9]. However, the polysaccharides from *Dendrobium fimhriatum Hook.var.oculatum Hook* have not been reported. Therefore, the purpose of the present investigation was to elucidate the isolation and characterization of water-soluble crude polysaccharide from the stems of *Dendrobium fimhriatum Hook.var.oculatum Hook*, as well as to evaluate its antioxidant activities *in vitro*.

## 2. Results and Discussion

### 2.1. Extraction of Water-Soluble Crude Polysaccharide

The polysaccharide, named DFHP, was obtained from the stem of *Dendrobium fimhriatum Hook.var.oculatum Hook* by the method of water-extraction and ethanol-precipitation. The total yield rate of the water-soluble polysaccharide was 18.64%. Because the molecular weight of polysaccharide was an important factor responsible for biological activities, determining the molecular weight was therefore the first step for the study of the polysaccharide. The molecular weight (M_w_) of DFHP was calculated to be 209.3 kDa based on the calibration curve obtained with standard dextrans.

### 2.2. Monosaccharide Composition of DFHP

**Figure 1:**
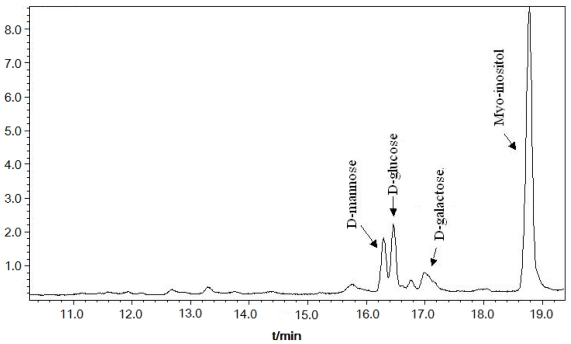
Monosaccharide compositions of DFHP by GC-MS analysis.

Monosaccharide composition of DFHP was analyzed by the trifluoroacetic acid hydrolysis and GC-MS analysis method. The results shown in Figure 1 indicate that d-glucose, d-mannose and d-galactose were the major monosaccharide constituents. The total content of monosaccharide compositions in DFHP was described as follows: mannose: glucose: galactose = 37.52%: 43.16%: 19.32%.

### 2.3. Infrared spectra of DFHP

**Figure 2:**
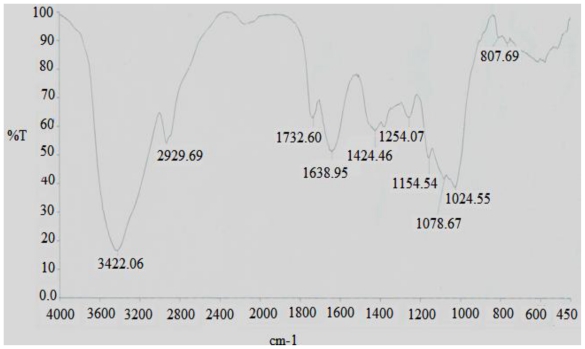
FT-IR spectra of the polysaccharide DFHP.

The FT-IR spectra of the DFHP were presented in Figure 2. The result exhibited a broad stretching intense characteristic peak at around 3422 cm^−1^ for the hydroxyl group[10], and a weak C–H band at around 2929 cm^−1^ [11]. The band at 1638 cm^−1^ was due to the bound water [12]. Also it has a specific band in the 1200–1000 cm^−1^ region, this region was dominated by ring vibrations overlapped with stretching vibrations of (C–OH) side groups and the (C–O–C) glycosidic band vibration [13]. Absorptions at 807 cm^−1^ were typical for α-dominating configuration [14].

### 2.4. NMR Identification of DFHP

**Figure 3:**
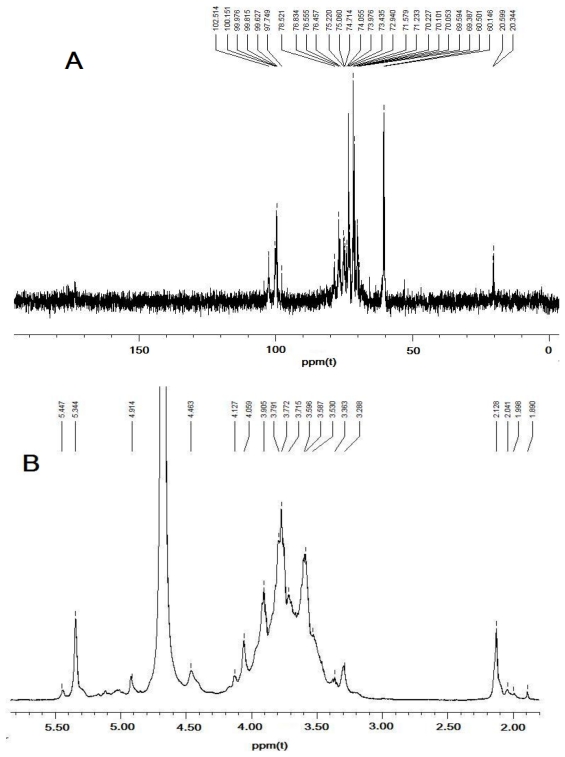
NMR analysis of DFHP. (**A**) for ^13^C-NMR analysis of DFHP, and (**B**) for ^1^H-NMR analysis of DFHP.

The ^1^H-NMR and ^13^C-NMR results for DFHP were assigned by comparison with the NMR data reported previously and detailed assignments of all signals are shown in Table 2 [15–18]. Based on previous discussions in the literature, chemical shifts between δ98 and 103 ppm are a typical feature of the C-1 in α-glycosidic linkages (Figure 3A), whereas in the case of β-glycosidic linkages signals would be expected between δ103 and 106 ppm [19,20]. Thus, the signals were centralized between δ99.63 and 102.51 ppm, indicating α anomeric configuration for all monosaccharide residues of DFHP. The ^1^H NMR spectrum of DDP showed two anomeric protons at 5.34 and 5.45, which were assigned as (1→4)-α-d-Glcp and (1→4)-α-d-Galp, respectively (Figure 3B).

### 2.5. Antioxidant Activities Analysis

#### 2.5.1. Effect of Scavenging DPPH Radicals

**Figure 4:**
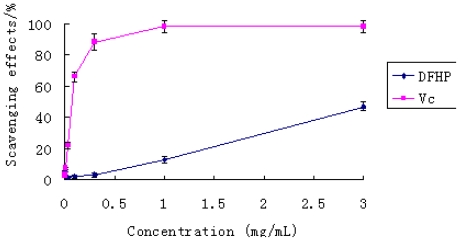
The scavenging effect of DFHP on DPPH radical. Results are presented as means ± standard deviations.

The DPPH free radical was a stable radical with a maximum absorption at 517 nm that can readily undergo scavenging by an antioxidant. So it has been widely accepted as a tool for evaluating the free radical scavenging activities of natural compounds. In the test, the scavenging ability of the polysaccharide DFHP on DPPH free radical was examined in the concentration range of 0.001–3.000 mg/mL. The result has been shown in Figure 4. From the figure, the activities of DFHP and Vc increased in a concentration dependent manner. Vitamin C exhibited very high radical scavenging activity at high doses (1.0–3.0 mg/mL). In contrast, the scavenging effect of the polysaccharide DFHP was lower than that of vitamin C at every dose; even at the high dose of 3.0 mg/mL, the scavenging activity was 46.67%. Therefore, DFHP has insufficient scavenging effect on DPPH radical scavenging.

#### 2.5.2. Scavenging Effects of Polysaccharide on Hydroxyl Radicals

**Figure 5:**
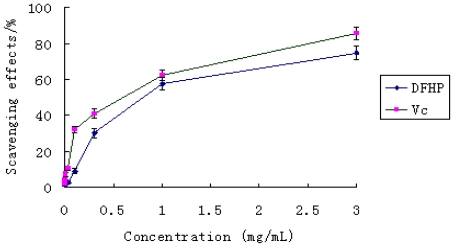
The scavenging effect of DFHP on hydroxyl radical. Results are presented as means ± standard deviations.

The scavenging ability of DFHP on hydroxyl free radical was shown in Figure 5. The two samples exhibited obvious hydroxyl radical scavenging activities in a concentration-dependent manner. The polysaccharide was found to have the ability to scavenge hydroxyl radicals at concentrations between 1.0 and 3.0 mg/mL. At 1.0 mg/mL, DFHP revealed an excellent hydroxyl radical scavenging activity (57.22%), similar to that of vitamin C (62.22%) (*P* < 0.05). At the high dose of 3.0 mg/mL, DFHP exhibited very strong activity (74.78%), which was close to the reference material vitamin C. These results clearly showed that DFHP has potential hydroxyl radical scavenging antioxidant ability.

#### 2.5.3. Scavenging Effects of Polysaccharide on ABTS

**Figure 6:**
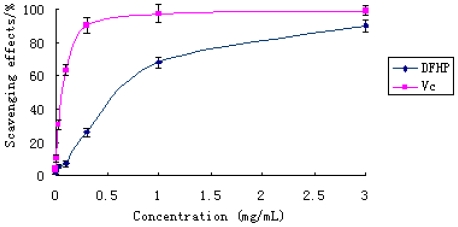
The scavenging effect of DFHP on ABTS radical. Results are presented as means ± standard deviations.

In the scavenging activity on ABTS assay, we employed a specific absorbance (734 nm) at a wavelength remote from the visible region in a short reaction time, and it can be used in both organic and aqueous solvent systems and can also be an index reflecting the antioxidant activity of the test samples [21]. The scavenging ability of DFHP on ABTS free radical was shown in Figure 6. The scavenging powers of DFHP and Vc correlated well with increasing concentrations. The reference material exhibited an excellent scavenging effect in high doses (from 0.3 to 3.0 mg/mL). At the same time, the polysaccharide DFHP also showed high scavenging effect on ABTS. Especially at 3.0 mg/mL, the effect reached 90.05%, which was close to that of vitamin C (P < 0.05). Therefore, the results indicated that DFHP had strong scavenging power for ABTS radicals and should be explored as novel potential antioxidants.

## 3. Experimental Section

### 3.1. Materials and Chemicals

DPPH (1,1-diphenyl-2-picrylhydrazyl) radical and Vitamin C were purchased from Sigma (St. Louis, MO, USA). Dextrans of different molecular weights were purchased from Pharmacia Co. (Uppsala, Sweden). The standard monosaccharides (glucose, mannose, rhamnose, galactose, xylose and arabinose) were purchased from the Chinese Institute for the Control of Pharmaceutical and Biological Products (Beijing, China). ABTS (2,2-azinobis-6-(3-ethylbenzothiazoline sulfonic acid) radical was purchased from Merck Co. (Darmstadt, Germany). Trifluoroacetic acid (TFA), pyridine, methanol, and acetic acid, ethanol, acetic anhydride and all other chemicals and reagents were analytical grade.

### 3.2. Preparation of the Polysaccharide from Dendrobium fimhriatum Hook.var.Oculatum Hook

Preparation of crude polysaccharide was carried out according to the method of Luo *et al.* [22], with some modifications. The stems of *Dendrobium fimhriatum Hook.var.oculatum Hook* were thoroughly washed with water, dried at 60 °C, and then powdered with a pulverizer. The powder of *Dendrobium fimhriatum Hook.var.oculatum Hook* was extracted successively with petroleum ether and ethanol, at first. After filtering, the residue was further extracted with double-distilled water at 100 °C for 2 h three times. Then all extracts were combined, concentrated and filtrated. The extract was deproteinized 5 times using the Sevag reagent [23], and the polysaccharide was free of proteins as scan by UV Spectra in 260 nm and 280 nm. After removal of the Sevag reagent, the extract was precipitated by adding ethanol (4 times the volume of aqueous extract), and the mixture was kept overnight at 4 °C for the polysaccharides. The precipitate was collected by centrifugation at 4000 rpm for 20 min, washed successively with petroleum ether, acetone and ethanol, the procedure of precipitation was perform iteration, and then dissolved in water and dialyzed against deionized water for 72 h, freeze-drying to yield the crude polysaccharide, which named DFHP.

### 3.3. Molecular Weight Determination

The molecular weights of polysaccharide were determined by Gel Permeation Chromatography (GPC) according to the method of Yamamoto *et al.* [24], in combination with a Waters HPLC (Waters 515, Massachusetts, USA) equipped with a Ultrahydrogel Linear Column (300 × 7.8 mm). The column was eluted with 0.2 M phosphate buffer (PH 7.0) at a flow rate of 0.7 mL/min and detected by a Waters 2410 Refractive index detector (RID). The detailed operation conditions were mobile phase: 0.2 M phosphate buffer (PH 7.0); flow rate: 0.7 mL/min; column temperature: room temperature; injection volume: 20 μL; running time: 20 min. Dextran standards with different molecular weights (2500, 4600, 7100, 10,000, 21,400, 41,100, 84,400, 133,800, 200,000 Da) were used for calibration curve.

### 3.4. Analysis of Monosaccharide Compositions

Identification and quantification of the monosaccharides of DFHP by using GC-MS, at first, 10.0 mg of DFHP hydrolyzed in a sealed glass tube with 2 M TFA (2 mL) at 100 °C for 8 h [25]. Then the hydrolysate was evaporated to dryness and dissolved in 0.5 mL of pyridine, after 10.0 mg hydroxylamine hydrochloride and 2.0 mg myo-inositol (as internal reference) were added to the solution, it was allowed to react at 90 °C for 30 min. The tube was cooled to room temperature, and then 0.5 mL of acetic anhydride was added and mixed thoroughly by vortexing. The tube was sealed and incubated in a water bath shaker set at 90 °C for 30 min. After cooled, approximately 1.0 μL of clear supernatant was loaded onto an Rtx-5SilMS column (30 m × 0.32 mm × 0.25 μm) of the GC-MS. Alditol acetates of authentic standards (glucose, mannose, rhamnose , galactose, xylose and arabinose) with myo-inositol as the internal standards were prepared and subjected to GC-MS analysis separately in the same way. The operation was performed in the following conditions: injection temperature: 240 °C; detector temperature: 240 °C; column temperature programmed: 160 °C holding for 2 min, then increasing to 240 °C at 5 °C/min and finally holding for 5 min at 240 °C. Nitrogen was used as the carrier gas and maintained at 1.0 mL/min.

### 3.5. Infrared Spectra of DFHP

The structural characteristics of the DFHP were determined by Fourier transform IR spectrophotometer (Perkin-Elmer Corp., USA). The purified polysaccharide DFHP was ground with KBr powder and then pressed into pellets for transform IR spectral measurement in the frequency rang of 4000–500 cm^−1^ [26].

### 3.6. NMR Identification

Twenty milligrams of DFHP was dissolved in D_2_O (0.5 mL, 99.9%), freeze-dried, and redissolved in D_2_O (0.5 mL). The ^1^H-NMR and ^13^C-NMR spectra were measured in an NMR tube (5 mm diameter) at 27 °C with a Bruker Avance 600 spectrometer. The chemical shift was expressed in parts per million (ppm).

### 3.7. Assays for Antioxidant Activities

#### 3.7.1. DPPH Radicals Scavenging Assay

In the present test, DPPH scavenging activities of the DFHP was measured according to the method of Shimada *et al.* [27] and Wei *et al.* [28], with some modifications. Vitamin C was used as reference material. Briefly, 0.1 mM solution of DPPH in methanol was prepared and 1.0 mL of this solution was added with 3.0 mL of the samples of various concentrations (0.001–3.000 mg/mL). The solution was kept at room temperature for 30 min, and the absorbance at 517 nm (A_517_) was measured. The DPPH scavenging effect was calculated as follows:

DPPH scavenging effect (%)=[Ao-(A-Ab)]/Ao×100

Where Ao: A_517_ of DPPH without sample, A: A_517_ of sample and DPPH, and Ab: A_517_ of sample without DPPH.

#### 3.7.2. Hydroxyl Radical Scavenging Assay

The hydroxyl radicals scavenging activity of the polysaccharide was measured according to the method of Wang *et al.* [29], with some modifications. Different concentrations (0.001–3.000 mg/mL) samples were incubated with 2.0 mmol/L EDTA-Fe (0.5 mL), 3% H_2_O_2_(1.0 mL) and 0.36 mg/mL crocus in 4.5 mL sodium phosphate buffer (150 mM, pH 7.4) for 30 min at 37 °C and hydroxyl radical was detected by monitoring absorbance at 520 nm. The hydroxyl radical scavenging effect was calculated as follows:

Hydroxyl radical scavenging effect (%)=[(Ac-As)/Ac]×100

Where As is the A_520_ of sample and Ac is the A_520_ of control, in the control, sample was substituted with distilled water, and sodium phosphate buffer replaced H_2_O_2_.

#### 3.7.3. ABTS Radicals Scavenging Assay

The radicals scavenging activity of DFHP against radical cation (ABTS^+^) was measured using the methods of Re *et al.* [30] and Luo *et al.* [31], with some modifications. ABTS^+^ was produced by reacting 7 mmol/L of ABTS^+^ solution with 2.45 mmol/L of potassium persulphate, and the mixture would be kept in the dark at room temperature for 16 h. In the moment of use, the ABTS^+^ solution was diluted with ethanol to an absorbance of 0.70 ± 0.02 at 734 nm. The sample (0.2 mL) with various concentrations (0.001–3.000 mg/mL) were added to 2 mL of ABTS^+^ solution and mixed vigorously. After reaction at room temperature for 6 min, the absorbance at 734 nm was measured. The ABTS^+^ scavenging effect was calculated by the following formula:

ABTS scavenging effect (%)=[Ao-(A-Ab)]/Ao×100

Where Ao: A_734_ of ABTS without sample, A: A_734_ of sample and ABTS, and Ab: A_734_ of sample without ABTS.

### 3.8. Statistical Analysis

The data were presented as mean ± standard deviation. Statistical analysis was conducted with the SPSS 16.0 software package.

## 4. Conclusions

In the present study, the polysaccharide DFHP(Mw 209.3 kDa) was first isolated from the stem of *Dendrobium fimhriatum Hook.var.oculatum Hook.* with the dominance of mannose, glucose, galactose. Free radicals scavenging activities *in vitro* indicated that DFHP has significant radicals scavenging abilities on ABTS and Hydroxyl radicals. The scavenging effects were powerful, and close to the positive control (Vc). Therefore, the polysaccharide DFHP should be explored as novel potential antioxidants. On the other hand, DFHP exhibited a weak scavenging effect on DPPH radical compared to the reference. Therefore, further investigation of its antioxidant activities *in vivo* would elucidate this further, and the detection of the mechanism of action relevant to its anti-oxidative activity will be carried out in future studies.
